# Dynamic surface behavouir and aqueous foam properties of graphene- polystyrene sulfonate / Cetyl trimethylammonium bromide mixtures

**DOI:** 10.1016/j.heliyon.2024.e41468

**Published:** 2024-12-24

**Authors:** Ali Akbar Ghofrani, Mahmoodreza Khadangi-Mahrood, Zahra Hejri, Susan Khosroyar

**Affiliations:** Department of Chemical Engineering, Quchan Branch, Islamic Azad University, Quchan, Iran

**Keywords:** PSS grafted graphene nanosheets, CTAB, Dynamic surface tension, Foamability, Foam stability

## Abstract

An interface can be delicately designed using interactions between nanoparticles and surfactants by controlling surface properties such as activity and charge equilibrium. This study seeks to provide insights into how surfactant concentration impacts the stability and dynamics of nanoparticle-surfactant interfaces, with potential applications in material science and interface engineering. This study investigates the interactions between Graphene Function (Gr, Graphene function in this text refers to functionalizing the graphene sheets with –COOH groups via acidic reactions.), Polystyrene sulfonate (PSS), and the surfactant Cetyl trimethylammonium bromide (CTAB) at the air/water interface. We examined various ratios of CTAB to Gr-PSS to determine the effects of surfactant concentration, focusing on conditions up to the critical micelle concentration (CMC). Specifically, we utilized different concentrations of CTAB ranging from 0 to 1 CMC (0.82 mM), while the concentration of Gr-PSS varied between 0 and 1 wt% and 20–50 ppm. To analyze the dynamic interfacial properties, including dynamic surface tension and dilational viscoelasticity, we employed drop profile analysis tensiometry (PAT) to measure area perturbation frequency at the air/water interface. The study aimed to elucidate the behavior of the CTAB/Gr-PSS complex at this interface. We discussed the adsorption of the CTAB/Gr-PSS complex on the droplet surface and its varying roles by examining surface pressure across different area change domains and conducting elasticity measurements. The results indicate that the attachment of CTAB molecules to Gr particles and PSS leads to the formation of surface-active complexes. As the surfactant concentration increases, excess CTAB monomers compete with the CTAB/Gr-PSS complexes for access to the interfaces, causing the larger complexes to migrate into the liquid bulk, as confirmed by elasticity assessments.

## 1 Introduction

Foams, inherently unstable dispersed systems from a thermodynamic standpoint, are a significant focus within colloidal chemistry. The study of foam stability, especially in the presence of solid particles like those found in three-phase or Pickering foams, has become increasingly important due to its implications for various technological processes. The choice of foam stabilizers depends on specific stability requirements and production conditions. Techniques such as gas bubbling and the incorporation of finely ground solids into the foam have been employed to enhance foam stability effectively [[1], [2], [3], [4], [5], [6]].

Theoretical postulations concerning the mechanisms of foam stabilization by solid particles encompass the formation of monolayers of bridging particles, bilayers of closely packed particles, and particle aggregates within the liquid film, all contingent upon the size of the particles in relation to the thickness of the liquid film [4,[7], [8], [9], [10]].

Liquid foams are utilized in a wide range of applications, including ore flotation, surfactant concentration and separation, fire extinguishing, and the production of heat-insulating materials and ceramics. The specific requirements for foam stabilization depend on their intended application, with stabilizers commonly consisting of surfactants and soluble polymers [3,[11], [12], [13]].

Several studies have been conducted on the interactions between nanoparticles, surfactants, and their mixtures at the interface between liquid and gas [1,4,10,[12], [13], [14]].

Some studies investigated the interactions and micellization properties of cetyltrimethylammonium bromide (CTAB) in the presence of sodium polystyrene sulfonate (NaPSS) and methyl red (MR) under varying conditions, including solvent composition and temperature. These studies key findings revealed that the critical aggregation concentration (CAC) and apparent critical micelle concentration (CMC) values decrease with increasing concentrations of NaPSS and MR, indicating enhanced micellization due to strong electrostatic interactions. Additionally, the introduction of ethanol affected micellization, with CMC∗ values rising in order of increasing ethanol volume fractions. Various thermodynamic parameters such as Gibbs free energy changes supported the feasibility and stability of the micellization process, particularly in ethanol-water mixtures. These researches highlighted the complex relationships between surfactants, polyelectrolytes, and dyes, offering insights into their potential applications in scientific and industrial fields [5,6,10,14,15].

Recent research suggests that cooperative behaviors and electrostatic interactions between surfactants and nanoparticles can enhance the stability of foams and emulsions. These interactions lead to increased adsorption of surfactants on nanoparticle surfaces, improving their hydrophobic properties. This study focuses on investigating the interactions between Gr and CTAB at the air/liquid interface using different ratios of surfactant to nanosheets [14,[16], [17], [18]]. The study examines mixtures of CTAB with small nanoparticles of Gr. By measuring the dynamic surface tension of these mixed systems at various molecular ratios, the study aims to understand the formation and behavior of the interfacial layer in response to low and high amplitude oscillations. The evaluation of dilational viscoelasticity in the mixed surfactant/nanoparticle films reveals how the structure of the complex film changes under small surface area perturbations. Higher frequency changes provide more detailed insights into the particles and complexes at the interface, enhancing our understanding of the structural behaviors and formation of macroscopic structures involving nanoparticles and surfactants [15,[19], [20], [21]].

In recent years, there has been a growing focus on developing foams stabilized with various solid particles, both with and without surfactant modifiers, leading to the emergence of so-called Pickering foams. These foams have demonstrated the ability to significantly reduce foam syneresis and gas transfer through diffusion, thereby markedly extending their lifespan [1,2,18,[22], [23], [24]].

This article seeks to investigate the foam-stabilizing effects of the cationic surfactant CTAB with Polystyrene sulfonate (PSS) grafted Graphene, showcasing the substantial foam-stabilizing capability of CTAB/Graphene–PSS composites attributed to the formation of surface-active complexes. Additionally, the study explores the influence of incorporating Graphene–PSS particles on the stability of foams derived from CTAB– Graphene–PSS composite foaming agents, with potential applications in various industrial and everyday settings.

## 2 Experimental

### 2.1 Materials

The natural graphite powder from Merck Company (USA), characterized by a purity exceeding 99.5 % and particle size below 50 μm, was acquired for the synthesis of functionalized Graphene. Polystyrene sulfonate (PSS) is a hydrophilic, linear polymer that creates copolymers of vinyl alcohol and vinyl acetate from Sigma-Aldrich (USA) has been used as it was received without further purification. Furthermore, CTAB from Merck Company (USA), boasting a purity level surpassing 98 %, as well as sulfuric acid (98 % H_2_SO_4_), hydrogen peroxide (30 % H_2_O_2_), hydrochloric acid (37 % HCl), and potassium permanganate (KMnO_4_) from Sigma-Aldrich (USA) were also obtained. All chemical substances were employed in their original state without undergoing any purification procedures.

### 2.2 Functionalized PSS- graphene synthesis

The method for synthesizing graphene was briefly outlined in literature [25,26]. In order to modify the surface of the nanoparticles, strong oxidizing agents such as sulfuric acid (H_2_SO_4_) and nitric acid (HNO_3_) were employed, as documented in reference [26]. Specifically, a blend of concentrated HNO_3_ and H_2_SO_4_ (in a 1:3 v/v ratio) was introduced to 1 g of nanoparticles, and the resulting solution was heated to 65 °C for 3 h. The resultant products were subsequently subjected to washing and filtration using distilled water until reaching a neutral pH level. Subsequently, the obtained sample was dried at 100 °C for 12 h.

### 2.3 Characterization

**Fig. 1:**
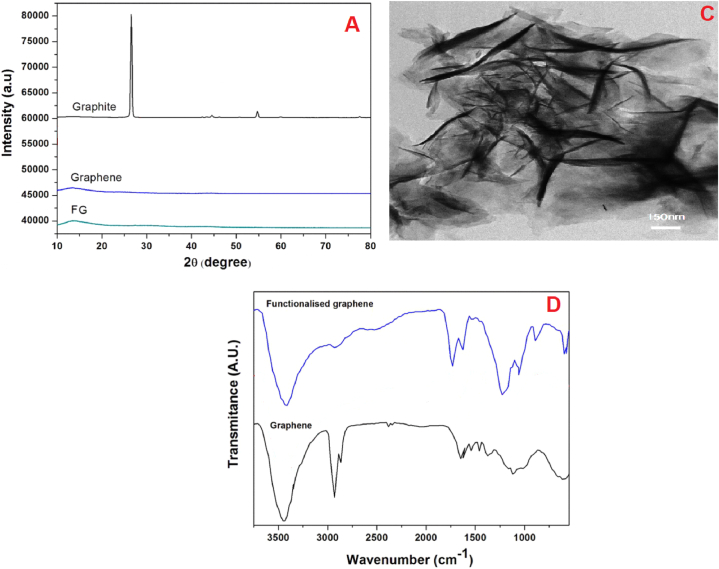
GR and Graphite XRD (A)- GR TEM image (C)- Graphene- GR FTIR (D).

Fourier Transform Infrared (FTIR) spectra were obtained using a PerkinElmer spectrophotometer from the United States, which encompassed a wavelength range spanning from 400 to 4000 cm^−1^. X-ray diffraction patterns (XRD) were captured utilizing an X'Pert MPD Philips instrument from Holland, equipped with CuKα radiation and operating at 40 kV and 30 mA. The size and morphology of FG were examined using Transmission Electron Microscopy (TEM) with a JEOL JEM-2010 F microscope operating at 200 kV. Dynamic interfacial tension measurements were conducted using a drop oscillation tensiometer (PAT1 instrument, SINTERFACE, Germany). A pendant drop with a fresh surface of dispersion was promptly generated at the stainless steel tip of a capillary by a computer-controlled pump and placed in a sealed cuvette [6,7]. Information regarding the foamability and foam stability of aqueous solutions was obtained through measurements based on the Ross-Miles technique. The Ross-Miles foam test was executed in accordance with ASTM D1173-07 (2007). Foams were generated through the process of allowing a 200 cm3 solution to descend from a predetermined distance of 90 cm into 50 cm3 of the identical solution. The height of the resultant foam was promptly measured upon the drainage of the pipette with the aid of a digital camera. Furthermore, the decrease in foam height over a period of time was also documented. The experiments were conducted four times, and the mean values of foam height were reported. All the characterization analyses are summarized in Fig. 1.

## 3 Results and discussion

### 3.1 Dynamic and equilibrium surface tension of PSS- graphene/CTAB mixtures

**Fig. 2:**
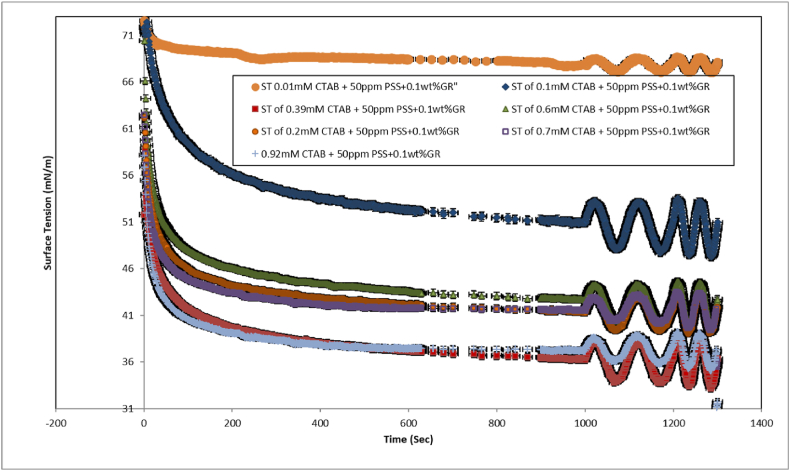
The dynamic surface tensions of 0.1 wt%GR+ 50 ppmPSS+ (0.01 mM–0.92 mM) CTAB system across concentrations ranging from 0 to 1 wt%. The surface tension measurements were taken during sinusoidal perturbations of drop surface area/volume, representing 7 percent of the initial drop volume after reaching equilibrium.

**Fig. 3:**
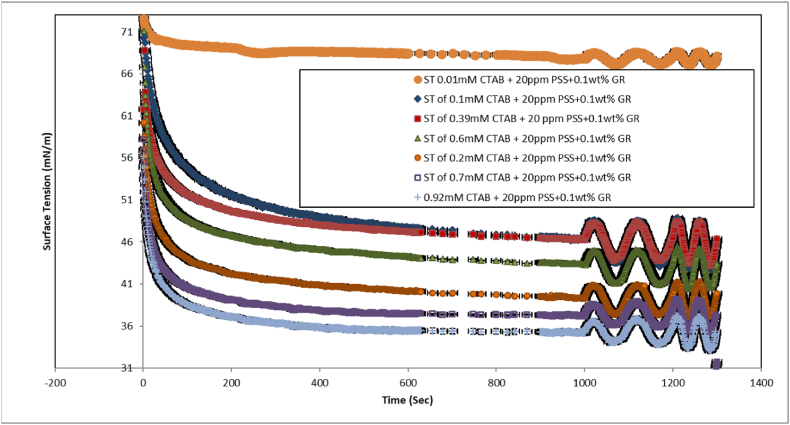
The dynamic surface tensions of 0.1 wt%GR+ 20 ppmPSS+ (0.01 mM–0.92 mM) CTAB system across concentrations ranging from 0 to 1 wt%. The surface tension measurements were taken during sinusoidal perturbations of drop surface area/volume, representing 7 percent of the initial drop volume after reaching equilibrium.

**Fig. 4:**
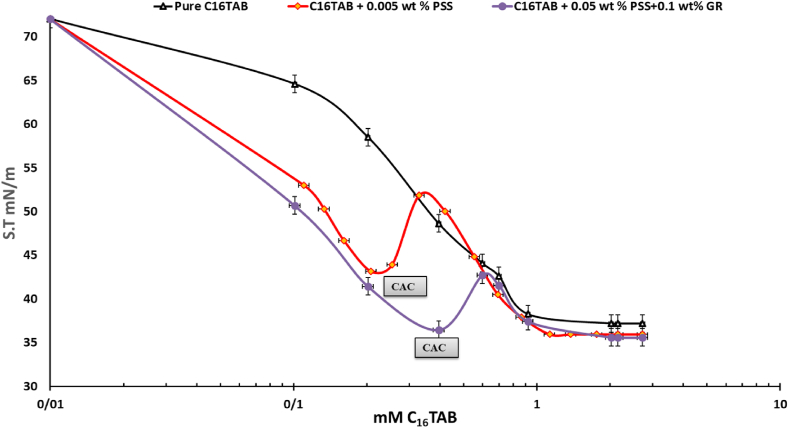
The Equilibrium surface tensions of pure CTAB, CTAB+50 ppm PSS and CTAB+0.1 wt% GR+50 ppm PSS with 0.01 mM–3 mM CTAB system across concentrations.The surface tension measurements were taken after sinusoidal perturbations of drop surface area/volume, representing 7 percent of the initial drop volume after reaching equilibrium. The C_16_TAB+0.002%wt PSS was also reported in Ref. [23] which is confirmed its results within this study.

**Fig. 5:**
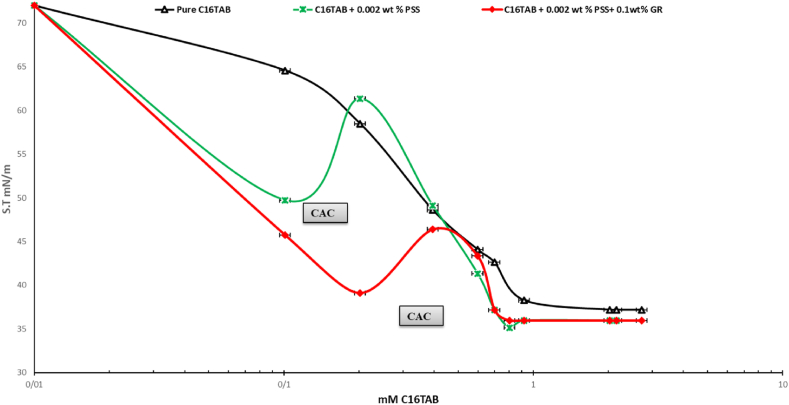
The Equilibrium surface tensions of pure CTAB, CTAB+20 ppm PSS and CTAB+0.1 wt% GR+50 ppm PSS with 0.01 mM–3 mM CTAB system across concentrations.The surface tension measurements were taken after sinusoidal perturbations of drop surface area/volume, representing 7 percent of the initial drop volume after reaching equilibrium.

Fig. 2, Fig. 3, Fig. 4, Fig. 5 illustrates the dynamic and equilibrium surface tension of GR dispersions at concentrations ranging from 0 to 0.1 wt %, measured after 1200 s with and without the addition of CTAB surfactant.

As illustrated in Fig. 4, this graph focuses on the dispersions of mixed GR at a concentration of 0.1 % by weight. The results indicate that as the concentration of CTAB increases, the surface pressure decreases, ultimately reaching a surface tension of 35 mN/m. This suggests that CTAB and the CTAB/GR-PSS complexes adsorb at the air-water interface. However, with a further increase in CTAB concentration, the surface tension continues to decline, indicating the formation of additional surface-active complexes. This decrease in surface tension is due to the high surface activity of CTAB and the migration of GR-CTAB and GR-PSS/CTAB complexes to the interface. The electrostatic repulsion and steric hindrance between the GR sheets and PSS molecules significantly influence the dispersion of CTAB, GR-CTAB, and GR-PSS/CTAB complexes. The conjugated structure of GR affects its hydrophilic and hydrophobic properties, leading to distinct non-polar characteristics that facilitate particle adsorption at the interface. It is important to note that the adsorption and desorption processes for GR-PSS/CTAB and GR nanoparticles occur more slowly than for surfactant molecules due to their larger size. As a result, the adsorption dynamics at the air-water interface for nanoparticle-containing systems are slower, meaning the initial and equilibrium surface tension values differ, this results confirmed by other researchers in a CTAB + PSS system [21,23,27]. As it can be seen the critical aggregation concentration (CAC) of the CTAB + PSS system in the 20 ppm and 50 ppm of PSS are 0.1 and 0.2 mM, respectively. By addition of 0.1 wt percent of the GR these CAC values turn out to be 0.2 and 0.4 mM and surface tension drops dramatically at these values (Fig. 5). These findings highlight that addition of GR nanoparticles activated further vacant sites for the adsrption of CTAB surfactant. In Refs. [[21], [22], [23]] Interactions between polymers and surfactants significantly discovered for foam formation and stability; however, the mechanisms behind these effects remain unclear and are currently under investigation. The Critical Aggregation Concentration (CAC) has been recognized as a key parameter in these studies, although it is primarily characterized as an equilibrium parameter. In contrast, the dynamics of adsorption play a crucial role in foam formation and stability. In similar research [21,23] focuses on the dynamic surface tension and viscoelastic properties of mixtures composed of Polystyrene Sulfonate (PSS), an anionic polyelectrolyte, and Cetyl Trimethyl Ammonium Bromide (CTAB), a cationic surfactant, at the air-water interface. The authors employed Profile Analysis Tensiometry (PAT) to gather data on these properties. The measured dynamic surface tension values reveal three distinct regions corresponding to different surfactant/polymer ratios. The findings indicate that a minimum number of surfactant molecules are necessary per polymer molecule for the polymer-surfactant complexes to exhibit sufficient surface activity and effectively adsorb at the water-air interface. At high surfactant concentrations, a surplus of CTAB competes with the polymer-surfactant complexes at the interface, resulting in the displacement of these complexes into the bulk solution, a phenomenon confirmed by our elasticity measurements.

At intermediate surfactant concentrations, where the complexes achieve peak surface activity, we observe rapid dynamics in adsorption, correlated with a peak in the measured elasticity values. Additionally, the results on foam formation and stability demonstrate a significant relationship with these observations, as evidenced by tests conducted using the Ross-Mill test and the gas sparging method.

### 3.2 Elasticity measurements

**Fig. 6:**
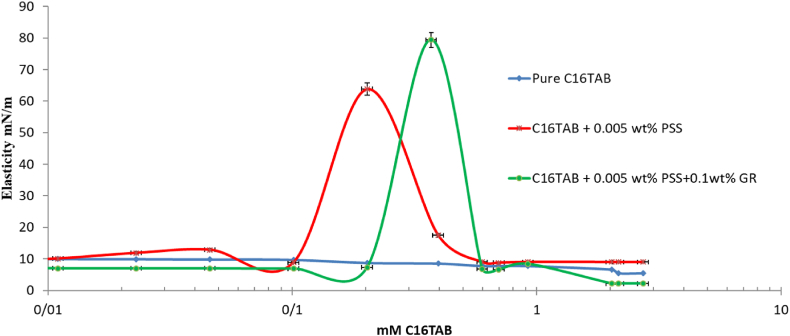
The Elasticity (at F = 0.02 Hz) of the pure CTAB, CTAB/50 ppmPSS and CTAB/0.1 wt%GR-50 ppm PSS as a function of the CTAB concentration. The C16TAB+0.002%wt PSS was also reported in Ref. [23] which is confirmed its results within this study.

The current investigation presents findings from an assessment of elasticity using drop oscillations (F = 0.02 Hz), as illustrated in Fig. 6, for GR concentrations of 0.1 wt% in conjunction with CTAB concentrations ranging from 0.1 to 3 CMC (0.82 mM) at 20 and 50 ppm of PSS.

**Fig. 7:**
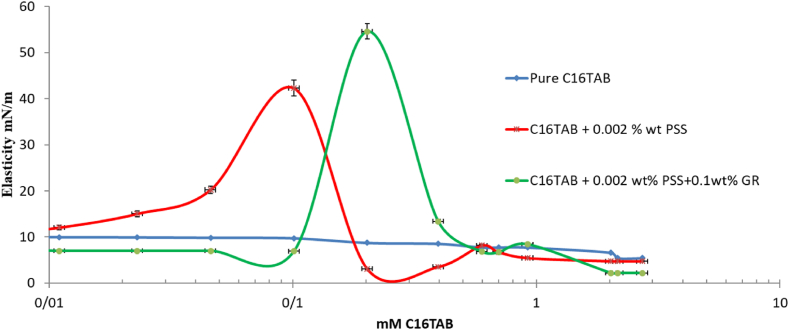
The Elasticity (at F = 0.02 Hz) of the pure CTAB, CTAB/20 ppmPSS and CTAB/0.1 wt%GR-20 ppm PSS as a function of the CTAB concentration. The C16TAB+0.002%wt PSS was also reported in Ref. [23] which is confirmed its results within this study.

The elasticity value demonstrates an increase for each sample of 0.1 wt% of GR within the 0.1 to 3 CMC range of CTAB, where the maximum adsorption of CTAB/PSS and CTAB/GR-PSS complex occurs. The delayed adsorption dynamics of large GR/CTAB and CTAB/GR-PSS complexes, as compared to that of a single CTAB molecule, are evident in Fig. 2, Fig. 3. The elevated elasticity values depicted in Fig. 6, Fig. 7 provide evidence of the presence of PSS/CTAB and CTAB/GR-PSS complexes on the surface at the CAC concentration, observed across all CTAB concentration ranges.

### 3.3 FG/CTAB foamability and foam stability

**Fig. 8:**
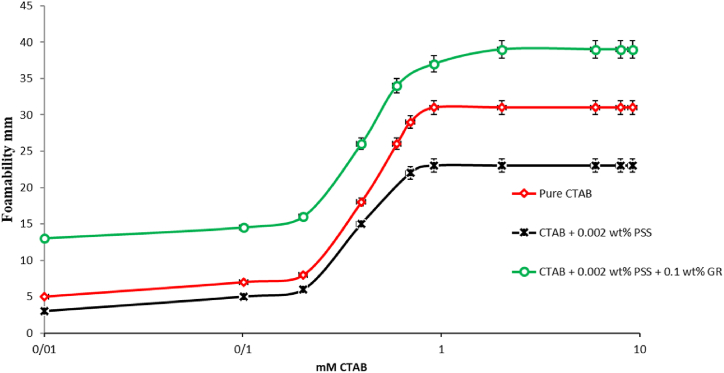
The Foamability of the pure CTAB, CTAB/20 ppmPSS and CTAB/0.1 wt%GR-20 ppm PSS as a function of the CTAB concentration.

**Fig. 9:**
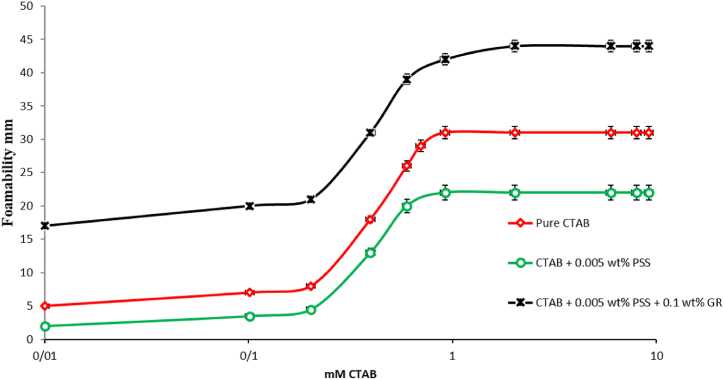
The Foamability of the pure CTAB, CTAB/50 ppmPSS and CTAB/0.1 wt%GR-50 ppm PSS as a function of the CTAB concentration.

The inherent incapacity of pure GR nanoparticles to form enduring foams necessitates the utilization of surfactants to facilitate foam formation. The foamability and stability of both pure CTAB, PSS/CTAB and GR-PSS/CTAB foams were evaluated using static foam testing. Fig. 8, Fig. 9 depicts the variations in foam height with CTAB concentration for 20 AND 50ppm-PSS/CTAB dispersions.

The repulsion force contributes to the migration of complexes toward the interface. These observations can be attributed to the rapid occurrence of bubble formation and liquid phase contact, which transpires in less than a second. Consequently, it can be inferred that the presence of GR nanoparticles, absence of free CTAB molecules, and inadequate time for PSS/CTAB complex adsorption onto the interface collectively result in diminished foamability. Adversly by addition of 0.1 wt% of GR the diminishing trend of foamability has been changed in a such a way that the system of CTAB+0.002/0.005 wt% PSS+0.1 wt% GR demonstrates higher foamability with respect to the CTAB+0.002/0.005 wt% PSS systems. This can be attributed to the competition between CTAB molecules and the other heavy complexes which result in higher surface activity of the CTAB monomers.

It was noted that the bubble diameter decreased as the relative concentration of PSS increased while keeping the GR concentration constant. The foam with the highest stability was distinguished by the smallest bubble size and increased surface elasticity. Furthermore, a greater concentration of PSS/CTAB structure led to the formation of thicker and more viscous lamella film, consequently resulting in the creation of more stable foam films.

**Fig. 10:**
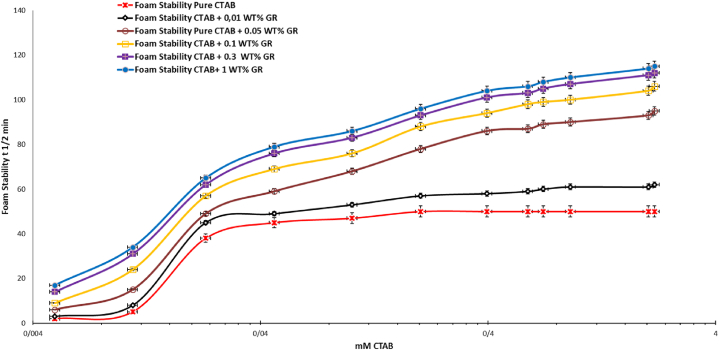
The Foam stability of the pure CTAB, CTAB/0.01 ot 0.1 wt%GR as a function of the CTAB concentration.

Fig. 10 illustrates the foam stability measurments of pure CTAB and GR/CTAB systems as it is clear by addition of further GR particles the foam stability enhanced dramatically as at 1 wt% of GR the foam stability doubled at all CTAB concentrations which can be attributed to the formation of thicker and more viscous lamella film.

**Fig. 11:**
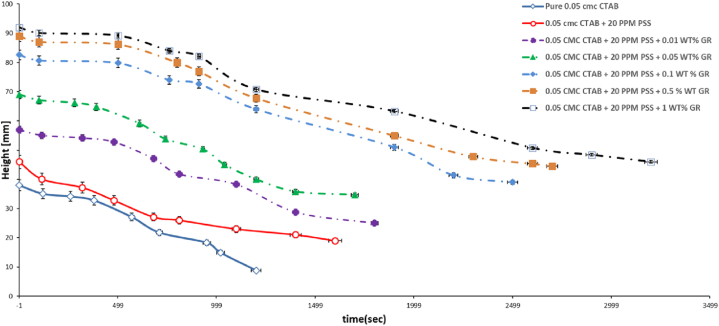
The Foam decay rate of the pure 0.05 mM CTAB, 0.05 mM CTAB/20 ppmPSS and 0.05 mM CTAB/20 ppmPSS – 0.01 to 1 wt% of GR as a function of the CTAB concentration.

Fig. 11 depics the foam decay rate of the pure 0.05 mM CTAB, 0.05 mM CTAB/20 ppmPSS and 0.05 mM CTAB/20 ppmPSS – 0.01 to 1 wt% of GR as a function of the CTAB concentration.

As it is noted the decay time which shows in somewhat the foam stability augmented as the 20 ppm of PSS added to the pure CTAB. By introducing various GR concentration by increasing GR concentration the decay time enhanced which lead to the higher foam stability. Again we have similar mechanism of foam stability which describes the formation of thicker and more viscous lamella film, consequently resulting in the creation of more stable foam films.

## 4 Conclusions

The primary aim of this investigation was to analyze the surface behavior of CTAB solutions containing PSS and GR nanoparticles, which function as amphiphilic agents, using techniques such as dynamic surface tension, foamability, and foam stability. The results showed that pure GR did not exhibit any tendency to adsorb at the air/water interface. The CTAB/PSS structures revealed varying levels of hydrophilicity at different CTAB concentrations, with CTAB adsorbing at the gas/liquid interface and competing with the surface of the CTAB/PSS complexes. This interaction between PSS and CTAB molecules resulted in improved foamability and foam stability while also affecting the adsorption dynamics at the critical aggregation concentration (CAC). Stabilized foams were created using GR aqueous suspensions combined with cationic CTAB/PSS in the hydrophobic regions of the CTAB/GR-PSS mixture. The findings suggest the existence of an additional CAC∗ point within the PSS/GR/CTAB system, which enhances the geometric structure of the foam and contributes to the distribution of bubble diameter, ultimately improving foam stability alongside elasticity and surface tension values. Overall, the results indicate that the specially designed binary and ternary systems are more effective at modifying surface properties compared to pure surfactants, demonstrating superior foaming capability. Additionally, these blends hold promise as effective wetting agents, making them particularly suitable for enhanced oil recovery (EOR) applications [2,8,[11], [12], [13],[16], [17], [18], [19], [20], [21], [22],^24^,[28], [29], [30], [31], [32], [33], [34], [35], [36], [37], [38], [39]].

## CRediT authorship contribution statement

**Ali Akbar Ghofrani:** Writing – original draft, Validation, Methodology, Investigation, Formal analysis, Data curation, Conceptualization. **Mahmoodreza Khadangi-Mahrood:** Writing – review & editing, Supervision, Methodology, Conceptualization. **Zahra Hejri:** Supervision, Methodology, Data curation, Conceptualization. **Susan Khosroyar:** Resources, Investigation, Formal analysis.

## Declaration of competing interest

The authors declare the following financial interests/personal relationships which may be considered as potential competing interests:

Mahmoodreza Khadangi reports article publishing charges, equipment, drugs, or supplies, and writing assistance were provided by Islamic Azad University Quchan Branch. Mahmoodreza Khadangi-Mahrood reports a relationship with 10.13039/501100002660Islamic Azad University Quchan Branch that includes: board membership, consulting or advisory, employment, and funding grants. If there are other authors, they declare that they have no known competing financial interests or personal relationships that could have appeared to influence the work reported in this paper.
